# Use of erythropoiesis-stimulating agents in children with chronic kidney disease: a systematic review

**DOI:** 10.1093/ckj/sfac058

**Published:** 2022-02-26

**Authors:** Gordon Bruce, Peter Schulga, Ben C Reynolds

**Affiliations:** Royal Hospital for Children Glasgow, Paediatric Nephrology, Glasgow, UK; Royal Hospital for Children Glasgow, Paediatric Nephrology, Glasgow, UK; Royal Hospital for Children Glasgow, Paediatric Nephrology, Glasgow, UK

**Keywords:** chronic renal failure, ESA, haemoglobin, paediatrics, systematic review

## Abstract

**Background:**

Erythropoiesis-stimulating agents (ESAs) revolutionized the management of anaemia in chronic kidney disease (CKD) when introduced in the late 1980s. A range of ESA types, preparations and administration modalities now exist, with newer agents requiring less frequent administration. Although systematic reviews and meta-analyses have been published in adults, no systematic review has been conducted investigating ESAs in children.

**Methods:**

The Preferred Reporting Items for Systematic Reviews and Meta-analyses statement for the conduct of systematic reviews was used. All available literature on outcomes relating to ESAs in children with CKD was sought. A search of the MEDLINE, CINAHL and Embase databases was conducted by two independent reviewers. Inclusion criteria were published trials in English, children with chronic and end-stage kidney disease and use of any ESA studied against any outcome measure. An assessment of risk of bias was carried out in all included randomized trials using the criteria from the Cochrane Handbook for Systematic Reviews of Interventions. Two tables were used for data extraction for randomized and observational studies. Study type, participants, inclusion criteria, case characteristics, follow-up duration, ESA type and dosage, interventions and outcomes were extracted by one author.

**Results:**

Of 965 identified articles, 58 were included covering 54 cohorts. Six were randomized trials and 48 were observational studies. A total of 38 studies assessed the efficacy of recombinant human erythropoietin (rHuEPO), 11 of darbepoetin alpha (DA) and 3 of continuous erythropoietin receptor activator (CERA), with 6 studies appraising secondary outcome measures exclusively. Recruitment to studies was a consistent challenge. The most common adverse effect was hypertension, although confounding effects often limited direct correlation. Two large cohort studies demonstrated a greater hazard of death independently associated with high ESA dose. Secondary outcome measures included quality of life measures, growth and nutrition, exercise capacity, injection site pain, cardiovascular function, intelligent quotient, evoked potentials and platelet function.

**Conclusions:**

All ESA preparations and modes of administration were efficacious, with evidence of harm at higher doses. Evidence supports individualizing treatments, with strong consideration given to alternate treatments in patients who appear resistant to ESA therapy. Further research should focus on randomized trials comparing the efficacy of different preparations, treatment options in apparently ESA-resistant cohorts and clarification of meaningful secondary outcomes to consolidate patient-relevant indices.

## INTRODUCTION

Chronic kidney disease (CKD) is a substantial global health burden, with mortality rates for children with end-stage kidney disease (ESKD) 55 times higher than the general paediatric population [1]. Anaemia is a common complication observed in up to 73% of children with CKD stage 3 and 93% in stages 4 and 5 [2, 3].

The primary cause of this anaemia is a deficiency of erythropoietin (EPO). EPO is a 30.4-kDa glycoprotein that stimulates red cell production, differentiation and survival [4]. EPO gene expression is upregulated by hypoxia-inducible transcription factor (HIF), although in CKD the response to hypoxia is deranged, resulting in impaired production and reduced HIF-binding capacity [5–7].

Erythropoiesis-stimulating agents (ESAs) replicate EPO. A recombinant human erythropoietin (rHuEPO) was synthesized in 1985 [8], trialled in 25 adults in 1987, with demonstrated efficacy [9].

The short half-life of rHuEPO necessitates administration three times per week [10]. In the late 1990s, darbepoetin alpha (DA) was synthesized through ‘glycoengineering’ amino acid changes to rHuEPO, extending its half-life to allow once- or twice-weekly dosing [11]. In 2007, continuous erythropoietin receptor activator (CERA) usage was approved, with the addition of a methoxy-polyethylene glycol polymer further prolonging the half-life to permit fortnightly or monthly dosing [12].

In adults, ESA therapy is associated with hypertension, stroke, vascular access thrombosis and overall mortality when higher haemoglobin (Hb) levels (>12.5 g/dL) are targeted [13, 14]. In children this association is less clear—one large retrospective cohort study of 1569 children found no relationship [15]. The Kidney Disease: Improving Global Outcomes (KDIGO) 2012 guidelines recommend modest Hb targets of 11.0–12.0 g/dL with initial doses of 60–150 IU/kg/week for rHuEPO and 0.45 µg/kg/week for DA [16]. There also appears to be an independent association with mortality when high ESA doses are administered [17, 18], therefore KDIGO specifically cautions against dose escalation in failed responders [16].

There are several large randomized controlled trials (RCTs) [19–21] feeding into systematic reviews appraising the efficacy of ESAs in adults [13, 22–24]. Although review articles exist [25–27], there are currently no systematic reviews regarding ESA use in children.

This systematic review will appraise studies assessing the efficacy of ESAs in children with CKD. It will also appraise the extent to which a safety profile has been established, while outlining all other secondary outcomes explored.

## METHODS

The Preferred Reporting Items for Systematic Reviews and Meta-Analyses (PRISMA) statement for the conduct of systematic reviews was used.

### Eligibility criteria

Published studies in English were included that investigated children with CKD using any ESA. Any outcomes were considered. Studies examining single-dose pharmacokinetics were excluded.

### Outcome measures

Primary outcome measures included any measure of red blood cell quantity and function. Secondary outcome measures included adverse effects and any other measure of physiological function or patient satisfaction.

### Search strategy

**Figure 1.**
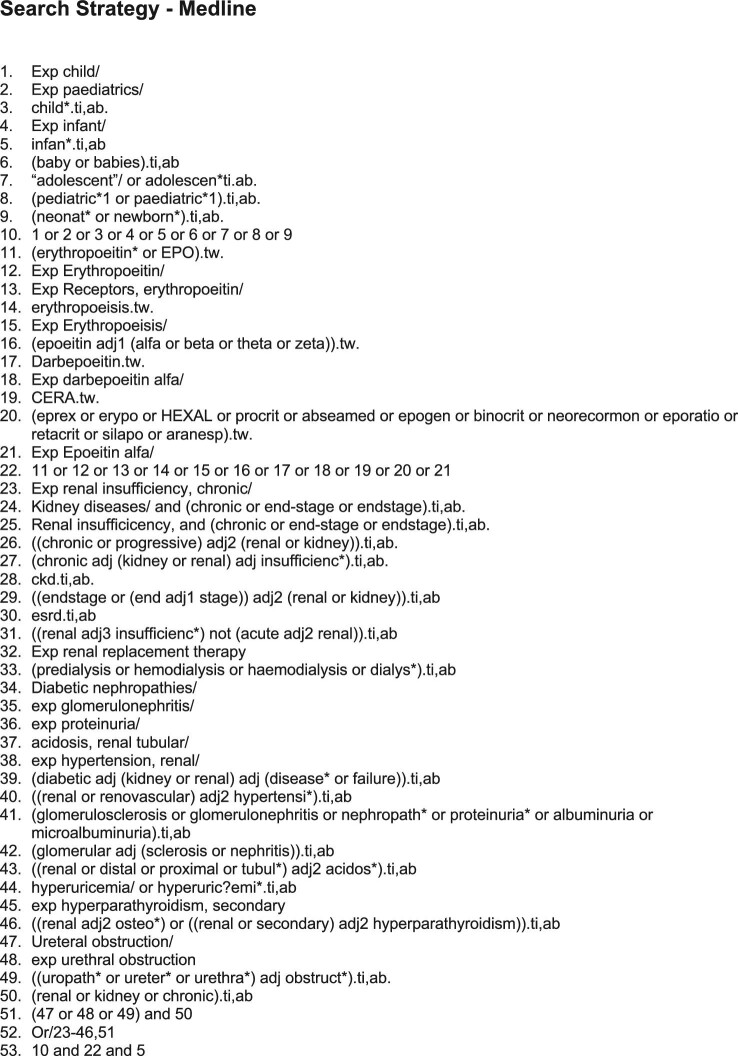
Literature search strategy.

A computerized search was undertaken using MEDLINE, Embase and CINAHL through February 2021 by two independent reviewers (see Figure 1). Years included in each search were 1946–2021, 1974–2021 and 1961–2021, respectively.

### Study selection

Both reviewers independently conducted a manual search. Titles and abstracts were assessed against inclusion criteria with duplicates and non-relevant studies removed. The remaining studies were reviewed in full. Studies pertaining to the same patient cohorts were collated.

### Assessment of risk of bias

An assessment of risk of bias was carried out on all included randomized trials using the criteria from the Cochrane Handbook for Systematic Reviews of Interventions [27].

### Data extraction

Two tables were used for data extraction for randomized and observational studies. Study type, participants, inclusion criteria, case characteristics, follow-up duration, ESA type and dosage, interventions and outcomes were extracted.

## RESULTS

**Figure 2.**
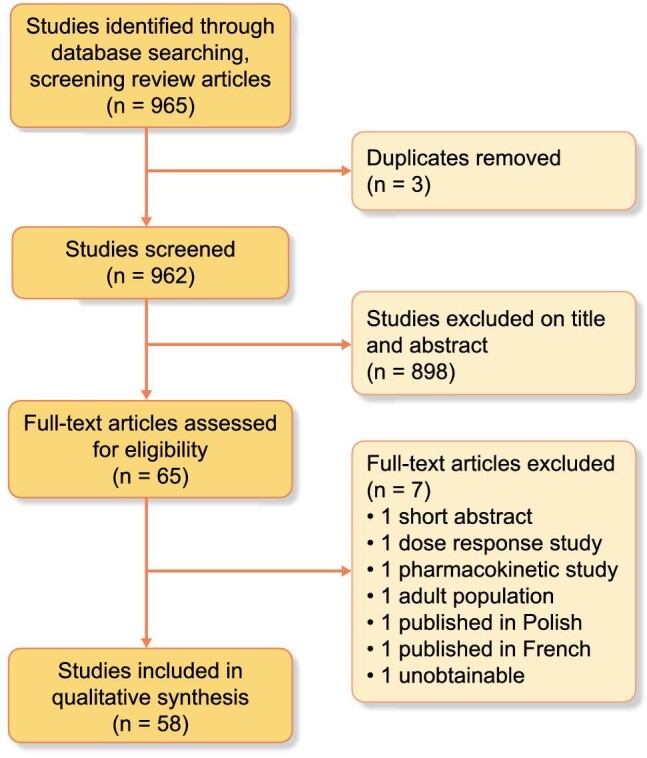
PRISMA flow chart.

The search identified 965 articles and 3 duplicates. A total of 898 articles were initially excluded and 65 studies were reviewed in full. A total of 7 studies were then excluded, leaving 58 studies included in the final review (see Figure 2). Collating studies with secondary analysis of identical cohorts resulted in 54 studies: 6 randomized trials and 48 observational studies. A total of 3895 children were included.

### Risk of bias summary

Risk of bias was conducted for six randomized trials (see Table 1). Randomization was low risk in one trial [28]. The other five were unclear risk, with missing details on randomization or concealment [29, 30]. Blinding was unclear risk in one study [30].

**Table 1.** Analysis of risk of bias

Risk of bias summary for randomized trials							
Study	Random sequence generation	Allocation concealment	Blinding	Incomplete outcome data	Selective reporting	Other bias	Justification
Warady *et al.* [29]	Unclear risk	Unclear risk	Low risk	Low risk	Low risk	Unclear risk	No details of randomization method or concealment
Schmitt *et al.* [58]	Unclear risk	Unclear risk	Low risk	Low risk	Low risk	Unclear risk	No details of randomization method or concealment
Warady *et al.* [28]	Low risk	Unclear risk	High risk	Unclear risk	Low risk	Unclear risk	No detail of concealment
Brandt *et al.* [30]	Unclear risk	Unclear risk	Unclear risk	Low risk	Unclear risk	Unclear risk	No details of randomization method or concealment No discussion around absence of blinding
Morris *et al.* [44, 45]	Unclear risk	Unclear risk	Low risk	Low risk	Unclear risk	Unclear risk	No details of randomization method or concealment Single blinded but unlikely to make difference to outcome
Yalçınkaya *et al.* [43]	Unclear risk	High risk	High risk	Low risk	Unclear risk	Unclear risk	No details of randomization method

### Characteristics of included trials

All trials had differences in study design, size, populations studied, interventions, outcomes and ESA investigated. Table 2 details six randomized trials. Table 3 details 48 observational studies.

**Table 2.** Characteristics of included randomized trials

Characteristics of included trials—randomized trials										
Author	Study design	Participants	Population	Inclusion criteria	ESA evaluated	Intervention	Control	Follow-up duration	Outcomes	Results
Warady *et al.* [29]	Prospective double-blinded RCT	114: 59 intervention, 57 control	43 centres: USA, European Union, Mexico	Age 1–18 years CKD CMT Hb <10 g/dL ESA naïve	DA sc/iv	Weekly (QW) dosing 0.45 µg/kg Adjustment increment not specified	Fortnightly (Q2W) dosing 0.75 µg/kg Adjustment increment not specified	24 weeks	Percent achieving target Hb (10–12 g/dL) Median time to target Hb QoL (PedsQL)	QW: 98% (>80% *P* < .001) Q2W: 84% (>80% *P* = .293) QW: 24 days Q2W: 22 days QW: 61.1 → 68.1 Q2W: 62.6 → 67.2
Schmitt *et al.* [58]	Prospective double-blinded RCT	13	Single-centre Germany	Age 3.7–22 years (mean 13.6) 10/13 PD 3/13 HD	DA sc 0.21–1.35 µg/kg/week rHuEPO Epoetin-beta sc 42–271 U/kg/week	DA then epoetin-beta injections	Epoetin-beta then DA injections	12 weeks	Mean pain perception (VAS 0–10)	DA Patient: 5.4 ± 1 Parent: 5.3 ± 1 Nurse: 4.4 ± 1 Epoetin-beta Patient: 2.3 ± 0.6 Parent: 2.0 ± 0.9 Nurse: 2.2 ± 0.6 *P* < .05 for all comparisons
Warady *et al.* [28]	Prospective randomized open- label non-inferiority trial	124: 82 intervention, 42 control	Multicentre (NOS) USA	Age 1–18 years CKD CMT Stable on rHuEpo >8 weeks Diastolic BP <95th centile	DA 100 U rHuEpo to 0.42 µg DA	DA (QW or Q2W) Adjustment increment not specified	rHuEPO Adjustment increment not specified	28 weeks	Adjusted mean ΔHb Mean % Hb values in target (10–12.5 g/dL) Change in dosing over time Safety	rHuEPO: −0.16 g/dL (95% CI −0.77–0.45) DA: 0.15 g/dL (95% CI −0.30–0.60) Difference: 0.22 g/dL (95% CI −0.47–0.92) rHuEPO: 73% DA: 75% rHuEPO: 55.9% required dose increase DA: 37.3% required dose increase rHuEPO:14% treatment-related adverse events DA: 20% treatment-related adverse events
Brandt *et al.* [30]	Prospective randomized open-label multiple-dose study	44 22 intervention 22 control	3 centres USA	Age <21 years CKD CMT 25/44 HD 9/44 PD ESA naïve Hb <−2SD for age BP <95th centile	rHuEPO iv 3/week	Low dose: 150 U/kg/week for 12 weeks or until target achieved	High dose: 450 U/kg/week for 12 weeks or until target achieved	12 weeks initial phase Up to 81 weeks dose adjustment (median 37 weeks)	% at target Hb (10–12 g/dL) in initial phase Mean time to target Mean rHuEPO dose Adverse events	High dose: 95% Low dose: 66% High dose: 5 weeks Low dose: 13 weeks 157 ± 107 U/kg per week HTN: 30% [high dose: 38% high dose, low dose 21% (*P* = .17)] Iron deficiency: 30%
Yalçınkaya *et al.* [43]	Prospective open-label multiple-dose study	20	Single centre Turkey	Age 5–16 years (mean 10) CAPD ESA naïve	rHuEPO sc 1–3/week	Low dose: 50 U/kg/week	High dose: 150 U/kg/week	6 months	Mean ΔHCT Mean ΔBP (MAP)	High dose: 0.19 → 0.32 L/L (*P* < .001) Low dose: 0.19 → 0.30 L/L (*P* < .001) High dose: 85 → 101 mmHg (*P* < .05) Low dose: 83 → 87 mmHg
Morris *et al.* [44, 45]	Prospective single-blind crossover trial	11	Single centre UK	Age 2.3–12.3 years (median 6.7) 1/11 HD 9/11 CAPD 1/11 CKD CMT ESA naïve	rHuEPO sc 1–2/week 50 U/kg/week then adjusted	Phase 1: rHuEPO Phase 2: Placebo	Phase 1: Placebo 3 Phase 2: rHuEPO	24 weeks per phase	Median ΔHb post-rHuEPO Dietary intake Anthropometric measures Exercise Tolerance (2 m walking distance) QoL Echocardiography	7.3 → 11.2 g/dL (*P* < .001) No significant changes No significant changes Increase (NOS) (*P* = .06) Improvement in two domains (self-created study) Reduced cardiac index (*P* = .01)

**Table 3.** Characteristics of included observational studies

Characteristics of included trials—observational studies, case reports and series								
Author	Study design	*n*	Population	Case characteristics	ESA	Duration	Outcomes measured	Results
Fischbach *et al.* [60]	Phase II, open-label, prospective, multiple-dose study	64	Multiple centres 10 countries, unspecified	Age 5–17 years (mean 12.6) CKD CMT Hb 10–12 g/dL On stable dose of rHuEPO or DA	**MPG-epo beta** (Mircera) iv 4 weekly **Group 1** (16/64): intermediate conversion factor **Group 2** (48/64): high conversion factor	20-week core phase 16-week dose adjustment 25% increments 1-year safety extension	Mean ∆Hb during evaluation phase (target 10–12 g/dL) % maintaining target Hb during evaluation phase Adverse effects	**Group 1:** 11.30 → 10.36 g/dL (95% CI 9.98–11.14) **Group 2:** 11.10 → 11.01 g/dL (95% CI 10.65–11.36) **Group 1:** 75% **Group 2:** 81% 7 worsening HTN 1 new HTN 4 vascular access thrombosis
Libudzic-Nowak *et al.* [56]	Retrospective case series	3	Single centre Switzerland	Age 1–7 months (mean 4 months) CKD CMT Hb 7.7–10.7 ESA naïve	**DA** sc fortnightly 0.27–0.5 µg/kg/week Adjusted in 25% increments	18–41 months	Mean ∆Hb (target 10.7–12 g/dL)	Target Hb achieved at 11–22 weeks
Gaydarova *et al.* [52]	Prospective case series	7	Single centre Hungary	Age 3–16 years CKD CMT 5/7 ESA naïve 2/7 rHuEPO	**DA** sc fortnightly 0.36 µg/kg/week Adjusted in 25–50% increments	5–34 months	% maintaining Hb (target >11.8 g/dL)	86%
Schaefer *et al.* [62]	Phase IV, prospective observational study	319	Multiple centres 13 EU countries	Age <16 years (mean 9.1)	**DA** Variable regimens	2 years	Adverse drug reactions Mean DA dose Mean baseline Hb Transfusion rate	Six events in four patients (1.3%) 1.4–2.0 µg/kg/month 11.1 g/dL 15% received one or more transfusions
Lestz *et al.* [18]	Retrospective cohort study	829	Multiple centres USA	Age <18 years (mean 12.9) HD/PD	**rHuEPO/DA** variable regimens	N/A	Adverse effects in relation to dose	Increased hazard of death in highest dose regimen versus reference [HR 3.37 (95% CI 1.37–8.26), *P* = .01] (EPO 100 to <200 units/kg/week, DA 0.49 to <1 µg/kg/week)
Borzych-Duzalka *et al.* [63]	Prospective cohort study	1394	Multiple centres Worldwide	Age 1 month–20 years (median 10.2) PD	**rHuEPO/DA** variable regimens	Up to 48 months Median 0.8 months	Adverse effects in relation to dose	Increased HR per 1000 IU/m^2^ per week, 1.33; *P* = .01
Can *et al.* [48]	Prospective case–control study	34	Multiple centres Turkey	Age 4–18 years (mean 11.4) Any renal disease including HD and PD On rHuEPO or DA for >6 months	**Group A: rHuEPO** IV/sc 50–150 U/kg 2–3/week **Group B: DA alpha** IV/sc 0.5 µg/kg weekly Adjusted in 25% increments	6 months	Mean ∆Hb (target 11–12 g/dL) Rate of change of Hb Injection site pain Adverse effects	**Group A:** 9.56 → 10.67 g/dL (*P* = 0.01) **Group B:** 9.19 → 10.35 g/dL (*P* = .02) No difference between Group A and B No difference between Group A and B Systolic HTN in one DA patient No significant difference in BP between groups
Hattori *et al.* [89]	Prospective case series	25	Single centre Japan	Age 1–18 years (mean 11.2) PD Stable on rHuEPO >8 weeks	**DA** iv 2–4 weekly 1 µg DA to 200 IU rHuEPO Adjustment increment not specified	28 weeks	Mean ∆Hb (Target 11–13 g/dL) % achieving target Hb	9.9 ± 1.0 → 11.1 ± 1.0 g/dL 88% (15 patients changed from 2 to 4 weekly dosing)
Jander *et al.* [59]	Cross-sectional study	117	Multiple centres Poland	Age 8–16 years (mean 13.8) HD and PD	**MPG-epo beta (7%)** **DA (19%)** **rHu EPO (74%)**	6 months	Mean EPO dose Mean Hb during observation period % with Hb >11 g/dL	99 U/kg/week 10.91 ± 1.18 g/dL 48%
Wedekin *et al.* [49]	Prospective case–control	12	Single centre Germany	Age 6–17 year (median 15.2) Post-renal transplant eGFR 17–73 mL/min/1.73 m^2^ 7/12 ESA naïve: cases 5/12 on DA: controls	**MPG-epo beta** iv 4 weekly 2.3 µg/kg/dose Adjustment increment not specified	6 months	Mean ∆Hb (target 11–12 g/dL) % achieving target Hb	**Cases**: 9.9 → 11.2 g/dL (*P* = .004) **Controls: **10.3 → 11.6 g/dL (*P* = .39) 9/12 (75%)
Cano *et al.* [61]	Prospective case series	16	Single centre Chile	Age 2–14 (mean 9.7) Hb >10 g/dL for >4 weeks PD On rHuEPO	**MPG-EPO beta** (Mircera) sc 2 weekly 0.5 µg/kg/dose Adjusted in 25/50% increments	6 months	Mean ∆Hb (target >11 g/dL) Dosing profile over time BP profile	Hb 11.12 → 12.2 g/dL 5/16 Hb >13 g/dL at end 3/16 switched to HD 2/16 transplanted 2/16 switched to once a month dosing Mean 57th centile (unchanged)
Andre *et al.* [51]	Prospective case–control	39	12 centres France	Age 11–18 (mean 15.2) CKD CMT HD/PD Pre-transplant 10/39 ESA naïve **(cases)** 29/39 on rHuEPO **(controls)**	**DA** sc 1–2 weekly 0.45 µg/kg/week 1 µg DA to 200 IU r-HuEPO Adjusted in 7%–24% increments	6 months	Mean ∆Hb (target 11–13 g/dL) % achieving target Hb Mean maintenance dose at end Adverse effects	**Cases:** 9.5 →11.7 g/dL **Controls:** 11.1 → 11.5 g/dL 66.7% (26/39) **Cases:** 0.34 µg/kg/week **Controls:** 0.73 µg/kg/week One vascular access thrombosis One abdominal pain
Rijk *et al.* [54]	Retrospective case–control	19	Two centres Netherlands	Age 0–17 years (mean 6.8) NIPD 11/19 ESA naïve **(cases)** 8/19 on ip rHuEPO **(controls)** %	**DA** ip 0.45 µg/kg/week 1 µg DA to 200 IU r-HuEPO Adjustment increment not specified	31.5 months (median)	Mean ∆Hb (target 10.9–12.8 g/dL) Median maintenance dose Peritonitis incidence Adverse effects	10.9 → 11.4 g/dL (**cases + controls)** 0.79 µg/kg/week **(cases + controls)** One episode every 25.1 months Three worsening HTNs
Boehm *et al.* [46]	Retrospective cohort study	47	Single centre Austria	Age 0.8–11.2 years (mean 6.0)	**Not stated**	2.5 years (median)	Likelihood for catch up growth observed >6 months (Odds Ratio) after EPO commenced	6.67 (*P* < .05)
Durkan *et al.* [55]	Retrospective case series	6	Single centre US	Age <1 year Weight <8 kg CKD CMT ESA naïve	**DA** iv weekly 0.45 µg/kg/week Adjusted in 25% increments	20 weeks	Mean ∆Hb (target 10–11 g/dL) % achieving target Hb Adverse effects	9.0 → 11.0 g/dL 50% (3/6) One pain at injection site
Geary *et al.* [53]	Prospective/retrospective case series	33	Single centre Canada	Age 1–18 years CKD CMT HD and PD ESA naïve/ on rHuEPO (not further specified)	**DA** sc weekly 0.45 µg/kg Adjusted in 30%–50% increments	28 weeks	Mean ∆Hb (target > 10 g/dL) % achieving target Hb Adverse effects	ESA naïve–9.0→ 11.6 g/dL Switched–10.5 →11.1 g/dL Combined: 10.2 →11.4 g/dL (*P* <.0001) 91% One new HTN DA more painful than rHuEPO in 57%
De Palo *et al.* [50]	Prospective case series	Seven	Single centre Italy	Age 7–15 years (mean 11.5) HD On rHuEPO (EPO alpha)	**DA** IV weekly 1.59 ±1.19 µg/kg/week (dose based on rHuEPO dose) Adjustment increment not specified	6 months	Mean ∆Hb (target 11–13 g/dL) Mean DA dose change over time to maintain target Hb Adverse effects	11.04 ± 1.53 → 11.44 ±1.14 g/dL 1.59 (SD ±1.19) → 0.55 (SD ± 0.14) µg/kg/dose (baseline–6 months) (*P* < .05) Suggested long-term dose 0.25–0.75 µg/kg/dose Two severe new HTNs One persistent elevation in platelets
Rusthoven *et al.* [39]	Prospective case series	20	Single centre Netherlands	Age 0.9–14 years (mean 3.8) CCPD ESA naïve	**rHuEPO** ip 3/week 200 units/kg/week 50 mL dialysis bag	12 months	Median ∆Hb (target 10.4–11.2 g/dL) Median dose to maintain target Hb Incidence of peritonitis Adverse effects	9.4 → 11.0 g/dL (range 8.96–13.1) 200 → 179 U/kg/week 1/11.2 person months None reported
Kausz *et al.* [38]	Prospective case series	14	Single centre US	Age 0.9–18 years (mean 7.9) CCPD On sc rHuEPO	**rHuEPO** ip 3/week 300 U/kg/week 50 mL dialysis bag	12 weeks	Mean ∆HCT Mean EPO dose sc versus ip Patient satisfaction Incidence of peritonitis Adverse effects	0.34 → 0.33 L/L (*P* > .05) **sc:** 279 ±126 U/kg/week **ip:** 290 ± 194 U/kg/week All patients preferred ip administration 1/32.5 person months [RR versus centre rates: 3.1 (95% CI 0.92–6.3)] One HTN
Port *et al.* [90]	Prospective case series	8	Single centre	Age 7–15 6/8 PD 2/8 CKD CMT ESA naïve	**rHuEPO** sc 1/week 100–170 U/kg/week	4–38 months	Increase in Hb (before treatment—end of monitoring)	2.5 → 5.6 g/dL (median 3.7)
Sieniawska and Roszkowska-Blaim [91]	Prospective case series	19	Single centre Poland	Age 4–17.5 years (mean 11.8) 11/19 on HD 8/19 on CAPD Off ESA for >8 weeks	**rHuEPO** sc weekly 50 U/kg/week For 12 weeks, then dose adjusted	24 weeks	Mean ∆Hb (target > 10 g/dL) % reaching target Hb at 12 weeks	**CAPD:** 7.7 ± 0.2 → 11.2 ± 0.6 g/dL (*P* < .001) **HD:** 7.7 ± 0.6 → 9.3 ± 0.8 g/dL (*P* < .001) **CAPD:** 100% **HD:** 64%
Reddingius *et al.* [42]	Prospective case series	10	Single centre Netherlands	Age 4.1–15.2 years (median 7.8) 8/9 CAPD 1/9 NIPD **Group A:** ESA naïve, 4/10 **Group B**: ip EPO, in 250-mL dialysis bag	**rHuEPO** ip 3/week 50 mL bag **Group A:** 300 U/kg/week **Group B:** Previous dose	6 months	Median ∆Hb (target 10.4–11.2 g/dL) Change in mean EPO dose to maintain Hb	**Group A:** 8.5 → 10.6 g/dL **Group B:** 10.9 → 10.9 g/dL **Group A:** 262 → 194 U/kg/week **Group B:** 266 → 234 U/kg/week
Steele and Vigneux [40]	Prospective case series	3	Single centre Canada	Age 11 months–11 years Two CCPD One CAPD On sc rHuEPO	**rHuEPO** ip 100–150 U/kg/week CCPD: 2/week direct injections with 20 mL dialysate CAPD: 2/week in 300-mL dialysate bag	6 months	Mean ∆Hb (target unspecified) Adverse effects	9.2–10.4 g/dL One incident of peritonitis
Burke [73]	Prospective case series	22	Multicentre Australia	Age 4 months–16 years (mean 9 years) 9/22 CKD CMT, 10/22 CAPD 1/22 CCPD 2/22 HD Hb <8 g/dL ESA Naïve	**rHuEPO alpha** sc 3/week Initial 100 U/kg/week, increased 50 U/kg/week each month if needed	12 months	Mean ∆Hb (target 9–11 g/dL) % reaching target Hb at 16 weeks Dose range required to maintain Hb Mean change in IQ	6.7 ± 0.7 → 9.6 ± 1.9 g/dL (*P* < .001) 90% 45–125 U/kg/week 92 ± 16.1 → 97.5 ± 17 (*P* = .007)
Van Damme-Lombaerts *et al.* [34]	Prospective case series	115	Multicentre France, Belgium, Switzerland	Age 0.5–20 years (median 11.6) HD ESA naïve	**rHuEPO** iv 2–3/week Initial 75 U/kg/week, increased 75 U/kg/week each month if required	12 months	Mean ∆Hb (target 9.6–11.2 g/dL) % reaching target Hb Median dose required to maintain Hb Quality of life Adverse effects	6.7 → 9.7 g/dL 81% **At target:** 150 U/kg/week **At 12 months:** 200 U/kg/week Mean score reflecting questionnaire assessing sleep/rest, alertness, feeling and daily activities: 10.79 → 11.84 [+10% (*P* < .05)] 20 new or worsened hypertension 15 thrombotic events
Morris *et al.* [47]	Prospective case–control	13	Single centre UK	Age 4.3–11 years 1/13 NIPD 1/13 HD 1/13 CKD CMT Group A: ESA naive Group B: stable Hb on RHuEPO	**rHuEPO** sc 3/week	12 months	Echocardiography	Group A: reduction in mean indices of LVH [left ventricular mass index (*P* = .02) and cardiothoracic ratio (*P* = .005)]
Ongkingco *et al.* [33]	Prospective case series	7	Single centre USA	Age 6.5–18.7 years (median 12.6) CCPD ESA naïve	**rHuEPO** sc 1–3/week **Induction:** 150 U/kg/week **Maintenance:** 8-week fixed dose 3/week 8-week adjusted dose 1/week	24 weeks	Mean ∆HCT Mean rHuEPO dose	0.20 → 0.32 (baseline → target achieved) 0.20 → 0.35 (baseline → end of 1/week maintenance period) (*P* = not significant) **3/week:** 85.7 ± 40.4 U/kg/week **1/week:** 87.0 ± 34.1 U/kg/week
Scharer *et al.* [65]	Prospective case series	11	Single centre Germany	Age 0.6–17 years CKD CMT ESA naïve	**rHuEPO** sc 3/week Initial 150 U/kg/week	13 months (mean)	Mean time to Hb target (11.5 g/dL) Mean EPO maintenance dose	72 days (18–203) 135 U/kg/week
Aufricht *et al.* [92]	Prospective case series	12	Single centre Austria	Age 0.8–12.5 years (mean 7.4) CAPD ESA naïve	**rHuEPO** **s**c 3/week Initial 100–120 U/kg/week	40 weeks	Mean ∆HCT (target 0.35–0.40) % on single dose/week therapy	0.24 (0.14–0.29)→0.40 (0.33–0.48) (*P* < .01) 80%
Montini *et al.* [93]	Prospective case series	24	Multiple centres Brazil, Italy	Age 0.3–18 years (mean 10.3) PD ESA naïve	**rHuEPO** sc 3/week Initial 50 U/kg/week Up to 300 U/kg/week	24 weeks	Mean ∆Hb Adverse effects	6.5 ± 1.4 → 9.4 ± 1.7 g/dL One severe worsening HTN
Martin *et al.* [71]	Prospective case series	18	Single centre USA	Age 3–20 years (mean 10.9) 15/18 CAPD 3/18 CKD CMT ESA naïve	**rHuEPO** iv/sc 150 U/kg/week then decreased to 75 U/kg/week once target HCT achieved	6 months	Mean ∆HCT (target 0.33) Echocardiography Exercise capacity (modified Bruce)	0.22 ± 0.03 → 0.33 ± 0.02 (*P* = .001) Unchanged during period, normal parameters See Table 4
Suppiej *et al.* [75]	Prospective case series with case control	14	Single centre Italy	Age 9–19 years (median 12.3) HD ESA naïve 10 healthy matched controls	**rHuEPO** iv 3/week	13 weeks (mean)	Evoked potentials (BAEP and MN-SSEP) Mean ∆Hb	**Peripheral:** BAEP: Mean wave I latency reduced in ESKD versus controls (*P* < .01), unaffected by anaemia correction. MN-SSEP: mean PCV wrist → Erbs point, N9 and N20 amplitude reduced in ESKD versus controls (*P* < .05), unaffected by anaemia correction. **Central**: BAEP: no difference versus controls 6.6 ± 0.9 → 10.9 ± 1.2 g/dL (*P* < .0001)
Sallay *et al.* [94]	Prospective case series	8	Single centre Hungary	Age 5.5–18 years (mean 12.2) 7/8 HD 1/8 CAPD ESA naïve	**rHuEPO** iv/sc Initial 160 U/kg/week up to maximum 400 U/kg/week	28 weeks	Mean ∆HCT (target 0.33) Dose range required to maintain Hb	0.18 → 0.33 250–300 U/kg/week
Hisano *et al.* [95]	Prospective multiple-dose case series	12	Single centre Japan	Age 2–18 years CAPD ESA naïve	**rHuEPO** iv 1–3/week **Group A** (8/12): 89 U/kg/week **Group B** (4/12): 260 U/kg/week for 8 weeks then 88 U/kg/week	24 weeks	Mean ∆HCT Adverse effects	**Group A:** HCT 0.19 ± 0.02 → 0.29 ± 0.02 **Group B:** HCT 0.18 ± 0.03 → 0.30 ± 0.04 Two in Group B new HTNs
Goldraich and Goldraich [32]	Prospective case series	6	Single centre Brazil	Age 0.5–15.8 years (mean 6) CAPD ESA naïve	**rHuEPO** sc 1/week 150 U/kg/week	12 weeks	Mean ∆Hb Adverse effects	6.6 ± 0.47 → 10.1 ± 1.2 g/dL 1 transient pain 1 pruritis
Campos and Garin [96]	Prospective case series	11	Single centre USA	Age 0.5–20 years (median 14) HD ESA naïve	**rHuEPO** IV 3/week Initial 150 U/kg/week Adjusted based on HCT	12 weeks	Mean ∆Hb Mean maintenance dose Adverse effects	6.2 g/dL ± 0.4 → 10 g/dL ± 0.3 142.5 ± 13.5 U/kg/week Two worsening HTNs Two new HTNs One clotted graft
Stefanidis *et al.* [67]	Prospective case series	10	Single centre Greece	Age 1.5–17 years (mean 9.1) CAPD ESA naïve	**rHuEPO** sc/iv 3/week 90–220 U/kg/week until target 9.5–10 g/dL achieved	1 year	Mean ∆ growth velocity Mean ∆ anthropometric measures (weight, mid arm circumference, triceps skin fold thickness)	No significant change after anaemia correction No significant change after anaemia correction
Reddingius *et al.* [41]	Prospective case series	16	Single centre Netherlands	Age 0.8–16.5 years (median 4.1) PD ESA naïve	**rHuEPO** ip 3/week Initial 300 U/kg/week	3–12 months	Mean ∆Hb (target 10.5–11.3 g/dL) Transfusion burden Mean final EPO dose	7.9 → 10.8 g/dL 22 transfusions in 6 months prior to study → no further transfusions 279 U/kg/week
Warady *et al.* [70]	Prospective case series/case–control: exercise capacity	9	Single centre USA	Age 7.8–17 years (mean 12.4) 8/9 APD 1/9 CAPD ESA naïve Five healthy age matched controls	**rHuEPO** sc 2/week Initial 100 U/kg/week Adjusted based on HCT	16 weeks	Mean ∆HCT Transfusion burden Exercise capacity Adverse effects	21.9 + 3.5% → 33.2 L/L + 3.1% 0.5 transfusions per patient-month → 0.05 transfusions per patient (*P* < .01). **See Table 4** Six reports of pain at injection sites
Ongkingco *et al.* [97]	Prospective case series	10	Single centre USA	Age 13 days–18.6 years (mean 10.5) CCPD ESA naïve	**rHuEPO** sc 3/week Initial 150 U/kg/week Adjusted based on HCT	11 weeks	% responsiveness to initial dose regimen (HCT increase of 0.05/week) Adverse effects	91% Two worsening HTNs
Hisano *et al.* [98]	Prospective case series	10	Single centre Japan	Age 2–18 years (mean 11.6) CAPD ESA naïve	**rHuEPO** sc/iv weekly 60–150 U/kg/week (mean 93 U/kg/week)	24 weeks	Mean ∆Hb	6.9 ± 0.8 → 9.4 ± 1.5 g/dL
Navarro *et al.* [99]	Prospective case series	23	Single centre Spain	Age 0.1–19 years 11/23 CKD CMT 7/23 CAPD 5/23 HD ESA naive	**rHuEPO** sc/iv Initial 50 U/kg/week	4.3 months (mean)	Mean ∆Hb (target 10–12 g/dL) Mean EPO dose Adverse effects	7.4 ± 1.3 → 10.7 ± 1.4 g/dL (*P* < .001) 289 ± 86 U/kg/week Four worsening HTNs
Scigalla *et al.* [35, 36]	Prospective case series	120	Multicentre Germany, France, Switzerland	Age 2–21 years (mean 13) 108/120 HD 12/120 CAPD ESA naïve	**rHuEPO** sc/iv Initial 120–300 U/kg/week	41 weeks (mean)	Mean ∆HCT Transfusion burden Median rHuEPO dose at 12 months Mean ∆SD score for height	0.19 → 0.30 L/L (start → last value) 103 transfusion dependent → 0 transfusion dependent 138 U/kg/week No change (start → last value)
Bianchetti *et al.* [100]	Prospective case series	18	Single centre Switzerland	Age 5–18 years (mean 12) HD ESA naïve	**rHuEPO** Epoetin alpha iv 2–3/week 75–300 U/kg/week (median 150 U/kg/week) Adjusted based on HCT	13–78 weeks	Median ∆HCT Adverse effects	0.17 ± 0.05 → 0.27 ± 0.02 Five worsening HTNs Three new HTNs One venous thrombosis
Rigden *et al.* [64, 66]	Prospective case series	6	Single centre UK	Age 3.9–15.8 years HD ESA naïve	**rHuEPO** iv 3/week Initial 30 U/kg/week Increased 2 weekly 75, 150, 300, 450 U/kg/week	24 weeks	Mean ∆Hb Mean time to target Hb (10–13 g/dL) % Responsiveness (increase in Hb NOS) Exercise tolerance Mean ∆ growth velocity Adverse effects	7.1 → 10.5 g/dL 11 weeks 100% See Table 4 Small improvement in pre-pubertal children (unquantified) One vascular thrombosis
Offner *et al.* [37]	Prospective case series	14	Single centre Germany	Age 5.9–22.1 years 4/14 CAPD 10/14 CCPD ESA naïve	**rHuEPO** intraperitoneal iv weekly 300 U/kg/week until HCT 0.3 then 100 U/kg/week	7.8 months (mean)	Mean ∆HCT Mean time to target HCT 0.3 Adverse effects	0.19 → 0.28 3.1 ± 1.7 months One worsening HTN Intraperitoneal administration stopped due to three incidents of peritonitis
Montini *et al.* [69, 72, 75]	Prospective case series with case–control: exercise capacity NS function	10	Single centre Italy	Age 2.5–18.75 years (median 11.8) HD ESA naïve	**rHuEPO** iv 3 weekly 75–150 U/kg/week	18 weeks	Mean ∆Hb Exercise tolerance Evoked potentials (BAEP and MN-SSEP) Mean platelet count Mean bleeding time	6.4 ± 0.9 → 11.5 ±1 g/dL **See Table 4** **Peripheral:** significantly longer in patients versus controls (*P* < .0001). Anaemia correction produced no modification. **Central:** prolonged interpeak latency 1/10–returned to normal with correction of anaemia. 236 ± 84 → 391 ± 157 × 10^9^/L (*P* < .05)
Sinai-Trieman *et al.* [31]	Prospective case series	5	Single centre USA	Age 12–18 years (mean 16.2) CCPD ESA naïve	**rHuEPO** sc 3 weekly 450 U/kg/week	5–8 months	Mean ∆HCT % Responsiveness (increase in HCT NOS) Transfusion burden	12.8 ± 3.1 → 8.2 ±3.2 s (*P* < .01) 0.22 ± 3 → 0.33 ± 1.9% (*P* < .001) 100% 5–18 transfusions → 0 transfusions

APD: automated peritoneal dialysis; CAPD: continuous ambulatory peritoneal dialysis; CCPD: continuous cycling peritoneal dialysis; CERA: continuous erythropoietin receptor activator; CMT: conservative management; DA: darbepoetin; EPO: erythropoietin; ESA: erythropoietin-stimulating agent; HCT: haematocrit; HD: haemodialysis; HTN: hypertension; iv: intravenous; NIPD: nightly intermittent peritoneal dialysis; NOS: not otherwise specified; PD: peritoneal dialysis; PCV: peripheral conduction velocity; rHuEPO: recombinant human erythropoietin; sc: subcutaneous; RR: respiratory rate; BAEP: brainstem auditory evoked potential; MNSEP: median nerve somatosensory evoked potentials.

### Primary outcome measure—efficacy

#### rHuEPO

A total of 34 studies evaluated rHuEPO efficacy in 673 children. Three were randomized trials, with the majority (*n* = 31) being prospective observational case series. A total of 16 studies included children on peritoneal dialysis (PD), 6 included children on haemodialysis (HD), 1 investigated conservatively managed CKD and 8 were mixed. A total of 28 observational studies evaluated efficacy, of which 22 evaluated subcutaneous or intravenous administration. All of these confirmed improvements in indices of anaemia with rHuEPO administration.

The first paediatric observational study in 1989 highlighted that Hb could be successfully maintained in five paediatric dialysis patients on subcutaneous treatment, reducing the requirement for transfusion and subsequent development of anti-human leucocyte antigen (HLA) antibodies [31]. At 450 U/kg/week, the dose used was three times the upper limit of current KDIGO recommendations, and three of the five patients developed worsening hypertension.

Two studies used fixed dose regimens, with the remainder titrating dosing [31, 32]. Initial doses ranged from 30 to 450 U/kg/week, with target haematocrit (HCT) ranging from 0.33 to 0.40 L/L and Hb from 9 to 13 g/dL. Dose frequency was usually three per week, although two studies explored weekly dosing. Goldraich and Goldraich [32] demonstrated the efficacy of once weekly 150 U/kg dosing in six children on continuous ambulatory PD (CAPD). Ongkingco *et al.* [33] found no significant decrease in HCT after 8 weeks, decreasing from thrice to once weekly maintenance dosing (with associated cost-benefit), although the study suffered from significant dropouts resulting in only seven recruits.

The majority of observational studies investigated small cohorts of between 5 and 24 children (mean 15), although two larger multicentre studies were conducted in 1991 and 1994 [34–36]. The earlier of these included 120 children across multiple European centres [35, 36], reporting a mean final dose requirement of 138 U/kg/week. The second recruited 115 ESA-naïve children treated with rHuEPO for up to 1 year [34]. A total of 81% achieved a target Hb of 9.6–11.2 g/dL, although 68% of ‘non-responders’ were transplanted earlier. The median maintenance dose for children <30 kg was 225 U/kg/week and 107 U/kg/week for children >30 kg.

Six observational studies investigated intraperitoneal administration [37–42]. The first trial by Offner *et al.* [37] was halted early due to a high rate of peritonitis. Subsequently, Reddingius *et al.* [41] trained parents to inject rHuEPO into overnight 20 mL/kg bags, demonstrating a reduced requirement for transfusion without an increased peritonitis incidence. Reddingius *et al.* [42] and Kausz *et al.* [38] demonstrated in small cohorts of 10 and 14 patients, respectively, that intraperitoneal administration could maintain Hb when switched from subcutaneous rHuEPO without a significant dose increase. Administration was via a 50-mL intraperitoneal daytime dwell and Reddingius *et al.* [42] also demonstrated a mean dose reduction with this method against a 250-mL prolonged dwell (266 → 234 U/kg/week). Kausz *et al.* [38] demonstrated a possible increased risk of peritonitis versus historical controls {respiratory rate versus centre rate: 3.1 [95% confidence interval (CI) 0.92–6.3]}.

The largest study was conducted by Rusthoven *et al.* [39], who followed 20 ESA-naïve children for up to 1 year after starting rHuEPO in three divided doses delivered in 50-mL bags. They were able to maintain target Hb levels with a modest dose of 179 U/kg/week and with a low peritonitis incidence of 1 per 11.2 patient-months.

Three studies were randomized trials [30, 43–45]. Morris *et al.* [44] undertook a single-blinded placebo-controlled randomized crossover trial in 11 ESA-naïve children, demonstrating a significant increase in the median Hb from 7.3 to 11.2 g/dL (P < .001). Yalçınkaya *et al.* [43] randomized 20 ESA-naïve children on CAPD to receive low- (50 U/kg/week) or high-dose (150 U/kg/week) rHuEPO for 6 months and found that while both doses were efficacious, the higher dose led to a statistically significant increase in the mean arterial BP from 85 to 101 mmHg. Four participants in the high-dose arm had to temporarily discontinue therapy due to uncontrolled hypertension, with two instances of hypertensive encephalopathy. Brandt *et al.* [30] randomized 44 children to low (150 U/kg/week) and high (450 U/kg/week) dosing for 12 weeks or until a 10 g/dL target Hb was reached. Attainment of the Hb target in the higher dose cohort was more rapid, though with a non-significant higher incidence of hypertension [high dose 38%, low dose 21% (P = .17)].

A further three studies examined secondary outcomes only and are outlined below [18, 46, 47].

#### DA

A total of 11 studies investigated DA efficacy in 411 children. There were two randomized trials and nine observational studies (five prospective case series, one retrospective case series, one pro- and retrospective case series, one prospective case–control, one retrospective case–control). Two included children on PD, one included children on HD, three included conservatively managed CKD and three were mixed. Two analyzed DA in ESA-naïve children, three included children established on an ESA and the remaining four included a mixture of naïve and ESA-treated children. All demonstrated that DA was efficacious in reaching a specified Hb target. Targets were varied and generally aimed for 11–13 g/dL, although only two studies matched their target to the KDIGO recommendation of 11–12 g/dL [48, 49]. Cohorts within the observational studies varied between 3 and 39 (mean 19) participants.

Dosing regimens and adjustment strategies varied in the observational studies. Initial dosing was reported between 0.27 and 1.59 µg/kg/week, with both weekly and fortnightly dosing trialled, although most starting doses were close to the KDIGO recommendation of 0.45 µg/kg/week. All studies titrated dosing.

The first observational study, conducted by De Palo *et al.* [50], recruited seven children titrated to intravenous DA from rHuEPO using a conversion factor (weekly epoetin alfa dose/200 = weekly DA dose). An initial mean dose of 1.59 ± 1.19 µg/kg coincided with two cases of hypertension with a rapid increase in Hb to >13 g/dL, necessitating intermittent discontinuation of treatment. The mean dosage at 3 months was 0.51 ± 0.18 µg/kg/week and the authors subsequently recommended a long-term dose of 0.25–0.75 µg/kg/week.

In a French multicentre study of 39 children, Andre *et al.* [51] reported an almost 2-fold higher mean dose requirement in patients switched to DA from rHuEPO as compared with ESA-naïve children [0.73 versus 0.34 µg/kg/week (P = .015)]. This was not replicated in other studies involving both ESA-naïve children and children on rHuEPO [52–54].

A prospective case–control study compared the efficacy of rHuEPO to DA [48]. Can *et al.* [48] split 34 children equally to receive rHuEPO 2–3/week or DA weekly and found no differences in the efficacy or adverse effects profile between either group.

Durkan *et al.* [55] and Libudzic-Nowak *et al.* [56] specifically investigated infants <1 year of age. Durkan *et al.* [55] found that only 50% of the six patients recruited reached target Hb levels of 10–11 g/dL despite a high mean administered dose of 1.2 µg/kg/week and normal iron studies. Libudzic-Nowak *et al.* [56] achieved target Hb concentrations of 10.7–12 g/dL in three infants ages 1, 4 and 7 months, but requiring doses of 0.3–0.7 µg/kg/week, generally higher than in older children.

One retrospective case–control study appraised intraperitoneal administration. Rijk *et al.* [54] evaluated 19 children, 8 of whom were previously on intraperitoneal rHuEPO. A high median dose of 0.79 µg/kg/week was required to sustain Hb levels at a mean of 11.5 ± 1.2 g/dL. Six cases dropped out due to transplantation, with a relatively low peritonitis incidence of one episode every 25.1 months.

Two randomized trials investigated DA efficacy [28, 29]. Warady *et al.* [28] conducted an open-label non-inferiority trial in 124 children randomized (1:2) to ongoing rHuEPO therapy or DA, with results demonstrating an equivalent mean change in Hb over 28 weeks. The same team performed a prospective, multicentre double-blind randomized controlled trial of 114 ESA-naïve children comparing weekly versus fortnightly titrated dosing [29]. This showed that the mean time to target Hb of 10–12 g/dL was equivalent (22 days and 24 days, respectively), although a greater proportion of patients on weekly dosing reached the target Hb at 24 weeks (98% versus 84%).

A further three studies evaluated secondary outcomes only and are discussed below [57–59].

#### CERA

No randomized trials were identified regarding CERA use in children. Three observational studies evaluated CERA in 92 children [49, 60, 61]. Cano *et al.* [61] studied 16 children over 6 months converted from rHuEPO to fortnightly subcutaneous CERA. They found Hb was maintained, although dosing varied significantly (0.5–2.9 µg/kg/dose). Wedekin *et al.* [49] conducted a prospective case series on 12 children after renal transplant using a monthly intravenous dosing regimen. After 6 months of follow-up, they demonstrated an increase in mean Hb in ESA-naïve patients and maintained Hb levels in patients switched from DA (although only 75% achieved a target of 11–12 g/dL). Fischbach *et al.* [60] conducted an open-label multicentre study on 64 children on stable ESA regimens. An intermediate conversion factor (4 mg every 4 weeks for each weekly dose of 250 IU epoetin alfa/beta or 1.1 mg DA) derived from adult studies was tested against a twice higher conversion factor over 40 weeks. The intermediate factor proved less adequate at maintaining stable Hb, with mean Hb dropping below the lower target threshold of 10 g/dL on several occasions, whereas the higher factor was associated with more stable target Hb levels.

### Secondary outcome measures

#### Safety

Most observational studies included a discussion of adverse effects, the most common being hypertension. Three studies specifically focussed on safety in large cohorts [18, 62, 63].

Borzych-Duzalka *et al.* [63] prospectively appraised the anaemia management of 1394 children on PD across 30 countries between 2007 and 2011 for up to 48 months. Of 1147 patients where the ESA dose was available, 2.1% with lower dose regimens (<6000 IU/m^2^/week) versus 5.3% with higher dose regimens (not specified) died (P = .02). Regression analysis demonstrated an independent increased risk of death on PD with higher ESA doses [hazard ratio (HR) per 1000 IU/m^2^/week 1.33; P < .01]. Children were more likely to be ESA sensitive with higher albumin levels, low serum parathyroid hormone and persisting diuresis.

Lestz *et al.* [18] conducted a retrospective cohort study using 12- to 18-month follow-up of mortality records linked to a US 2005 ESKD registry in 820 children on dialysis and ESA therapy who had not undergone transplantation during 12–18 months of follow-up. Over the observation period, 60 children (7%) died, primarily attributed to cardiovascular causes. ESAs were prescribed to 95% of survivors and 93% of those who died. Average ESA doses were significantly higher in those who died versus survivors [rHuEPO 502 versus 290 units/kg/week (P < .001), DA 0.59 versus 2.6 µg/kg/week (P < .001)] and multivariate analysis demonstrated an HR of death of 3.37 in a high-dose group (EPO ≥350 units/kg/week or DA ≥1.5 µg/kg/week) when compared with a lower reference range (EPO 100–<200 units/kg/week or DA 0.49–1.0 µg/kg/week). This finding was independent of a wide range of factors, including cause of ESKD, dialysis modality, access and achievement of a minimum target Hb level of 11 g/dL.

Schaefer *et al.* [62] conducted an observational registry study of 319 children across 37 centres, the most comprehensive study of the safety of DA in children. Children were followed for up to 2 years, although 176 children withdrew earlier. A total of 162 patients, 50.8% of the cohort, reported a total of 434 serious adverse events (SAEs), the most common of which were peritonitis (*n* = 32), gastroenteritis (*n* = 19) and hypertension (*n* = 13). The authors state that this is comparable with a general cohort of children with CKD.

Four patients (1.3%) suffered six documented serious adverse drug reactions (SADRs): arteriovenous fistula thrombosis, priapism, thrombocytopenia, haemolysis, haemolytic anaemia and partial blindness. The authors suggest the latter four SADRs had more plausible explanations than related to ESA administration. Six fatal adverse events occurred, but none were considered to be related to ESA administration. No new safety issues were identified.

Two studies primarily focussed on efficacy also included safety extensions to their trials. Warady *et al.*'s [28] open-label non-inferiority study of DA versus rHuEPO included documentation of adverse events deemed by the investigator to be treatment related, affecting 14% (*n* = 6) of the rHuEPO cohort and 20% (*n* = 16) of the DA cohort. Injection site pain was the most common adverse event [12% (*n* = 5) rHuEPO, 11% (*n* = 9) DA], with hypertension in three of the DA cohort, one instance of vascular access thrombosis in both cohorts and access stenosis in one in the DA cohort.

Fischbach *et al.* [57] included a 1-year safety extension to their trial of CERA, including 37 children. It found no additional safety signals, with two SAEs, both vascular access thromboses. Hypertension was reported as an adverse event in 13% [60].

#### Quality of life (QoL)

Three studies assessed QoL [29, 34, 45]. Two studies used a non-validated self-designed questionnaire in children on rHuEPO. Small patient numbers for analysis in the first study (*n* = 7) prevented meaningful conclusions, while the second lacked any control arm but did demonstrate an improved QoL from baseline [34, 45]. Warady *et al.* [29] used the Pediatric Quality of Life Inventory (PedsQL) score to assess changes in QoL in their RCT cohort of 114 children starting DA. The authors noted a statistically significant increase in the PedsQL score from baseline to 6 months (QW: 61.1 → 68.1, Q2W: 62.6 → 67.2).

#### Growth and nutrition

**Figure 3.**
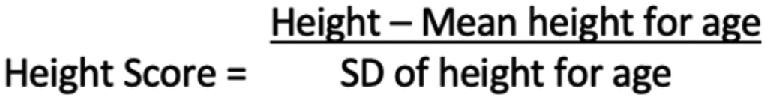
Height score (Scigalla 1991 [35]).

Five papers studied aspects of growth. Scigalla *et al.* [35] employed a height score (see Figure 3), finding no significant changes. Two studies assessed small cohorts of six participants [64–66]. Rees *et al.* [66], analysing Rigden *et al.*'s [64] 1990 cohort of six children on HD, described small improvements in growth velocity in the three youngest children over 1 year, with no appreciable effect in older participants. Scharer *et al.* [65] noted improvements in height standard deviation scores in the two youngest children in their cohort over ∼1 year of rHuEPO therapy [−1.8 → −1.0 and −3.7 → −2.5 standard deviation score], with minimal changes in four older children.

Stefanidis *et al.* [67] found no significant change in growth in 10 children 1 year after anaemia correction. These papers were summarized as the subject of a 1996 review [68]. Subsequently, Boehm *et al.* [46] conducted a retrospective cohort study in 47 children followed from initial referral to pre-dialysis care and after the initiation of dialysis. They reported that rHuEPO therapy initiation at referral was the only modifiable factor independently associated with a catch-up growth velocity once dialysis was initiated {odds ratio (OR) 6.67 [95% confidence interval (CI) 1.00–44.10], P < .05}.

#### Exercise capacity

Five studies investigated exercise capacity using treadmill tests (see Table 4). Baraldi *et al.* [69] demonstrated improvements in several domains using an unspecified treadmill protocol 2–4 weeks following anaemia correction in seven children. Rigden *et al.* [64] demonstrated an improved treadmill time using the modified Bruce protocol in four children on HD. Warady *et al.* [70] assessed nine children undergoing PD, demonstrating improvements in all parameters using the Balke protocol 1 month following achievement of the target HCT of 30%. It was the only study that used controls, comparing results with five age-matched children without renal disease and confirming a significant improvement in children with renal disease. Martin *et al.* [71] found mild sustained improvements in treadmill time in 12 children. Morris *et al.* [45] included exercise testing, although the results were unpublished.

**Table 4.** Assessments of exercise capacity

	*Martin et al.* [71]		*Warady et al.* [70]				*Baraldi et al.* [69]			*Rigden et al.* [64]	
	Modified Bruce		CMH Max/Balke				Not specified			Modified Bruce	
Protocol	A	B	A	Controls A	B	Controls B	A	B	Controls	A	B
VO_2_ (mL/kg/min)	26.4 ± 4.1	25.1 ± 5.4	17.8 ± 5.2	40.8 ± 12.3	24.0 ± 7.6*	42.0 ± 12.4	24.1 ± 7.1	32.6 ±12.7**	44.7 ± 7.1		
VO_2_AT (mL/kg/min)			13.1 ± 3.9	29.4 ± 6.3	17.1 ± 3.5*	28.2 ± 8.4	17.6 ± 6.3	25.9 ± 8.1**	31.4 ± 3.1		
Treadmill time (min)	10.3	12.1****	5.5 ± 1.3	8.7 ± 2.8	7.9 ± 1.5*	9.4 ± 3.0				13.4	16.8***

A: initial evaluation; B: second evaluation; C: VO_2_AT, oxygen consumption at anaerobic threshold; VO_2_: peak oxygen consumption. *P < .05% patient versus control; **P < .05 B versus A; ***P < .02 B versus A; ****P = 0.001 B versus A.

#### Injection site pain

Schmitt *et al.* [58] conducted a double-blinded RCT with 13 children assigned to receive DA injections followed by rHuEPO or vice versa, demonstrating a statistically significant increase in subjective pain with DA.

#### Cardiovascular function

Four studies assessed cardiovascular function. Montini *et al.* [72] and Martin *et al.* [71] assessed echocardiographic changes after anaemia correction, finding no significant changes. Morris *et al.* [44] demonstrated a reduction in cardiac index with rHuEPO versus placebo. Morris *et al.* [47] also compared seven ESA-naïve children to those established on ESAs, demonstrating improvements in cardiothoracic ratio and left ventricular mass.

#### Other secondary outcome measures

Infrequently considered outcome measures included intelligence quotient (*n* = 1) [73], platelet function (*n* = 2) [71, 73] and evoked potentials (*n* = 1) [71, 74].

## DISCUSSION

ESAs have transformed the management of renal anaemia, reducing transfusion burden and HLA sensitization. They are widely used in the USA and European Union, where up to 94% of children on HD are prescribed a regular ESA [76, 77]. Yet challenges remain—the European Dialysis Transplant Association registry reported in 2012 that 33.4% of children on dialysis <2 years of age and 31.2% >2 years had Hb levels below target [77].

This systematic review identifies a highly heterogeneous collection of studies assessing the use of ESAs in children. The challenges of recruiting within a paediatric cohort were apparent, with larger datasets requiring the involvement of multiple centres across countries.

Early studies of rHuEPO were characterized by small prospective observational cohorts demonstrating efficacy whether given subcutaneously or intravenously, while identifying that higher doses were associated with adverse events such as hypertension and vascular thrombosis. ESAs were shown to be less effective in the presence of iron deficiency and most subsequent studies ensured adequate iron stores.

A number of other secondary measures were explored using rHuEPO, varying from patient-relevant measures such as exercise tolerance and quality of life to physiological parameters, including cardiac function, evoked potentials, growth and nutrition, and platelet function. These were conducted on small cohorts.

The randomized placebo–controlled crossover trial and case–control studies conducted by Morris *et al.* [44, 47] suggest improvements in cardiac function following anaemia correction. Transplant recipients established on ESAs demonstrate comparably more minor cardiovascular improvements following transplantation when compared with CKD patients. This suggests that anaemia rather than uraemia correction plays a greater role in improving cardiac health or that other factors may be more important post-transplantation [47].

Studies on DA generally featured larger cohorts demonstrating non-inferiority against rHuEPO and established a similar safety profile [28, 62]. Weekly and fortnightly dosing both appear feasible treatment options [29]. QoL was explored in one study [29]. A modest increase in the PedsQL score was noted after 6 months of treatment, a finding supported by larger cross-sectional studies that demonstrate improved QoL in children with CKD without anaemia compared with those with persistent anaemia [78]. Further interrogation of outcomes relevant to patients has not been forthcoming. This review concurs with a Cochrane review of 2014 that noted that ‘formulations based on patient centred outcomes … are sparse and poorly reported’ [22]. More studies incorporating patient-centred outcomes are required to strengthen the rationale for intervention and choice of agent.

Early studies on CERA demonstrated efficacy in small paediatric cohorts and that Hb could be maintained when switching from other ESA preparations [60]. A higher conversion factor than that used in adults when changing from other ESA preparations may be required, and the safety profile appears similar to other ESAs [18]. Randomized trials comparing dosing regimens, comparing CERA with other ESAs and comparing patient preferences are lacking. A further dosing study is currently under way [79].

Intraperitoneal administration was predominantly evaluated using rHuEPO. It appears feasible and safe and is supported by pharmacokinetic studies demonstrating comparable bioavailability to other routes [80–82]. Nevertheless, it remains an uncommon route of administration. Intraperitoneal DA appears non-inferior to intraperitoneal rHuEPO, although only one study was identified.

Small studies on infants demonstrate that particularly high ESA doses may be required [55, 56]. Larger observational studies have also demonstrated higher ESA dose requirements in younger cohorts that appear consistent across ESA types [16, 34, 63]. One suggested reason for this apparent ESA resistance is a greater prevalence of iron deficiency: a study of anaemia in 2899 children on dialysis enrolled in the United States Renal Data System between 1996 and 2000 found that children ages 0–4 years were least likely to achieve target Hb, correlating with the lowest use of intravenous iron (33.9% versus 71%, ages 15–19) [83]. In contrast to this, Borzych-Duzalka *et al.*’s [63] study of 1394 children enrolled in the International Paediatric Peritoneal Dialysis Network registry between 2007 and 2011 found no relationship between Hb levels and iron supplementation, with an inverse association between Hb and ferritin levels (although transferrin saturation data were not available).

This suggests other mechanisms may contribute to an apparent ESA resistance in younger children. Speculated causes include higher numbers of EPO receptors that do not contribute to erythropoiesis, potentially ‘mopping up’ ESAs and reducing their haematopoietic potential [84]. Borzych-Duzalka *et al.* [63] also found reduced dose discrepancies in younger children when weight was substituted for body surface area (BSA) as a metric, suggesting requirements may be more proportional to metabolic rate than weight-based data suggest. Further studies that compared body weight with BSA dosing may help confirm this finding. Other studies have identified markers of dialysis adequacy, indices of nutritional intake, inflammatory status and hyperparathyroidism as primary factors in determining ESA resistance rather than iron deficiency [85, 86].

Nevertheless, the consistent finding of an independent relationship between higher ESA doses and mortality is of concern [18, 63]. High doses of ESAs can directly cause endothelial damage, vasoconstriction and platelet activation, all of which could plausibly increase the risk of cardiovascular mortality in children [87, 88]. Although the observational nature of the studies in question prevents the establishment of a definitive causal link, caution should clearly be applied when titrating ESAs in clinical practice, with careful consideration of all available interventions to maximize haemoglobin.

The most common reported adverse effect was hypertension. While some individual cases were clearly attributable to very high doses of ESAs [50, 74], in general the rate of hypertension in observational studies was noted to be comparable with other CKD cohorts.

Overall, there is no evidence to recommend one ESA as more efficacious or safe than any other. Factors influencing the decision of which ESA to choose will depend on considering the most convenient means of administration, taking into account age, mode of renal replacement therapy (if any) and patient preference. The morbidity and mortality risks associated with greater dosages of ESAs mandate thorough assessment of children with apparent ESA insensitivity.
